# 3-D stacked polycrystalline-silicon-MOSFET-based capacitorless DRAM with superior immunity to grain-boundary’s influence

**DOI:** 10.1038/s41598-022-18682-y

**Published:** 2022-08-24

**Authors:** Sang Ho Lee, Jin Park, So Ra Min, Geon Uk Kim, Jaewon Jang, Jin-Hyuk Bae, Sin-Hyung Lee, In Man Kang

**Affiliations:** School of Electronic and Electrical Engineering, Kyungpook National University, Daegu, 702-201 South Korea

**Keywords:** Engineering, Nanoscience and technology

## Abstract

In this paper, a capacitorless one-transistor dynamic random access memory (1 T-DRAM) based on a polycrystalline silicon (poly-Si) metal-oxide-semiconductor field-effect transistor with the asymmetric dual-gate (ADG) structure is designed and analyzed through a technology computer-aided design (TCAD) simulation. A poly-Si thin film was used within the device due to its low fabrication cost and feasibility in high-density three-dimensional (3-D) memory arrays. We studied the transfer characteristics and memory performances of the single-layer ADG 1 T-DRAMs and the 3-D stacked ADG 1 T-DRAMs and analyze the reliability depending on the location and the number of grain-boundaries (GBs). The relative standard deviation (RSD) of the threshold voltages (*V*th) is depending on the location and the number of GBs. The RSDs of the single-layer ADG 1 T-DRAM and the 3-D stacked ADG 1 T-DRAM are 1.58% and 0.68%, respectively. The RSDs of retention time representing the memory performances are 54.7% and 41%, respectively. As a result of the 3-D stacked structure, the averaging effect occurs, which greatly aids in improving the reliability of the memory performances as well as the transfer characteristics of 1 T-DRAMs depending on the influence of GBs. The proposed 3-D stacked ADG 1 T-DRAM helps implement a high-reliability single-cell memory device.

## Introduction

Recently, due to the complexity of the conventional DRAM’s capacitor fabrication, the 1T-DRAM has attracted great attention as a replacement for the conventional DRAM. The 1T-DRAM operates without the use of external capacitors, instead of relying on the floating body effect. The 1T-DRAM has the advantages of being easy to manufacture and having excellent logic device compatibility^1–5^. However, the smaller sizes of these devices tend to limit their retention characteristics due to the stronger electric field between the body and the source/drain junction. The stronger electric fields accelerate the recombination/generation process of the excess holes, resulting in shorter RTs in scaling of the 1T-DRAM. Various groups have conducted many studies, and the ADG structure can be a great solution to overcome the RT of 1T-DRAMs^5^. In addition, the 3-D stacked 1T-DRAM is made of polycrystalline silicon (poly-Si), so high-density 3-D memory arrays can be feasible. Based on their superior advantages in terms of the integrated fabrication technology, the poly-Si-based transistors have been widely used in 3-D memory technology in the past^6–8^.

The thermal budget was one of the significant challenges in implementing 3-D stacked transistors. The second layer and beyond of the device require high-temperature processing, which can threaten existing metallization materials or cause dopant diffusion in the lower layers of the device^9^. These difficulties in the fabrication can be overcome by using excimer laser crystallization (ELC) and it can implement the 3-D stacked 1T-DRAM. Fabrication steps are outlined in references 4,10 and 11. The key fabrication steps for the proposed 3-D stacked 1T-DRAM are summarized in more detail in the Supplementary Information. ELC can solve the thermal budget issues, however, it cannot solve the random distribution of the GBs. Because they randomly varied depending on the laser irradiation energy density^10^. The GBs are important in transistors made of poly-Si because they directly affect the transistor’s electrical performances. An analysis of the effect of GBs in a single channel was carried out in reference 5. However, a statistical study on the effect of the GBs in 3-D stacked ADG 1T-DRAM based on MOSFET with poly-Si has not been reported yet.

In this work, the 3-D stacked ADG poly-Si MOSFET based 1T-DRAM with various average grain sizes (*G*_*size*_s) cells are investigated. A TCAD simulation is used to demonstrate the superior reliability of 3-D stacked ADG 1T-DRAM to the effects of the GBs^11^. The proposed 3-D stacked ADG 1T-DRAM cells’ transfer characteristics, as well as memory performances, are analyzed and compared with the single-layer ADG 1T-DRAM. It has been proven to have excellent reliability to the effects of GBs.

## Simulation methodology

### Device structure

**Figure 1:**
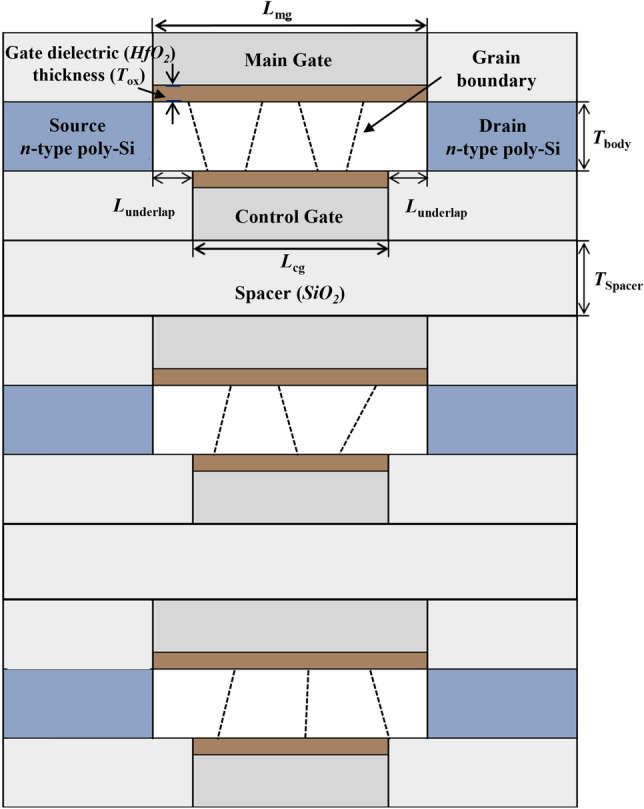
The cross-sectional view of the proposed 3-D stacked ADG poly-Si MOSFET-based 1 T-DRAM cell with an ADG structure.

Figure 1 shows the cross-sectional view of the 3-D stacked ADG poly-Si-MOSFET-based 1T-DRAM cell with an ADG structure to implement high-reliability GB-independent electrical characteristics and memory performances. The main gates are used to control the conventional MOSFET operation during the read period and the tunneling operation during the program period. The control gates located below the channel regions are used to operate the tunneling phenomenon during program operation. Additionally, they sweep out holes during the erase period, helping to maintain the stored holes during the hold operation. Each layer’s main gates share a common electrode, likewise the control gates share a common electrode. We refer to the operating bias of 1T-DRAM in reference 5. The work-functions of the main gate (*WF*_*MG*_) and the control gate (*WF*_*CG*_) are 4.85 eV and 5.3 eV, respectively. The main gate length (*L*_mg_), the control gate length (*L*_cg_), the underlap length (*L*_underlap_), the body thicknesses (*T*_body_), the gate dielectric (*HfO*_2_) thicknesses (*T*_ox_) and spacer thicknesses (*T*_spacer_) are 70 nm, 50 nm, 10 nm, 12 nm, 3 nm, and 30 nm, respectively. The spacer is made of silicon oxide (*SiO*_2_), a low-*κ* material that reduces parasitic capacitance and leakage components between channels. The doping concentrations of the source, body, and drain regions are 1 × 10^20^ *cm*^*−*3^ (n-type), 1 × 10^18^ cm^−3^ (p-type) and 1 × 10^20^ cm^−3^ (n-type), respectively. The values of the work-functions of the gates, the length of the gate, the underlap length, etc., refer to reference 5. Variations of underlap length in the proposed device are summarized in more detail in the Supplementary Information. Table 1 summarized the device parameters for each of the proposed devices.

**Table 1:** Device parameters of the proposed 3-D stacked ADG 1 T-DRAM used for the simulation work.

Parameter	Value
Main gate length (*L*_*mg*_)	70 nm
Control gate length (*L*_*cg*_)	50 nm
Underlap length (*L*_*underlap*_)	10 nm
Body thicknesses (*T*_*body*_)	12 nm
Gate dielectric (*HfO*_2_) thicknesses (*T*_*ox*_)	3 nm
Spacer (*SiO*_2_) thicknesses (*T*_*spacer*_)	30 nm
Average grain size (*G*_*size*_)	30 nm
The SD of *G*_*size*_	10 nm
Source/Drain doping concentration	n-type, 1 × 10^20^ cm^−3^
Body doping concentration	p-type, 1 × 10^18^ cm^−3^
Main gate work-function (*WF*_*MG*_)	4.85 eV
Control gate work-function (*W F*_*CG*_)	5.3 eV

### Physical models for accurate simulation works

**Figure 2:**
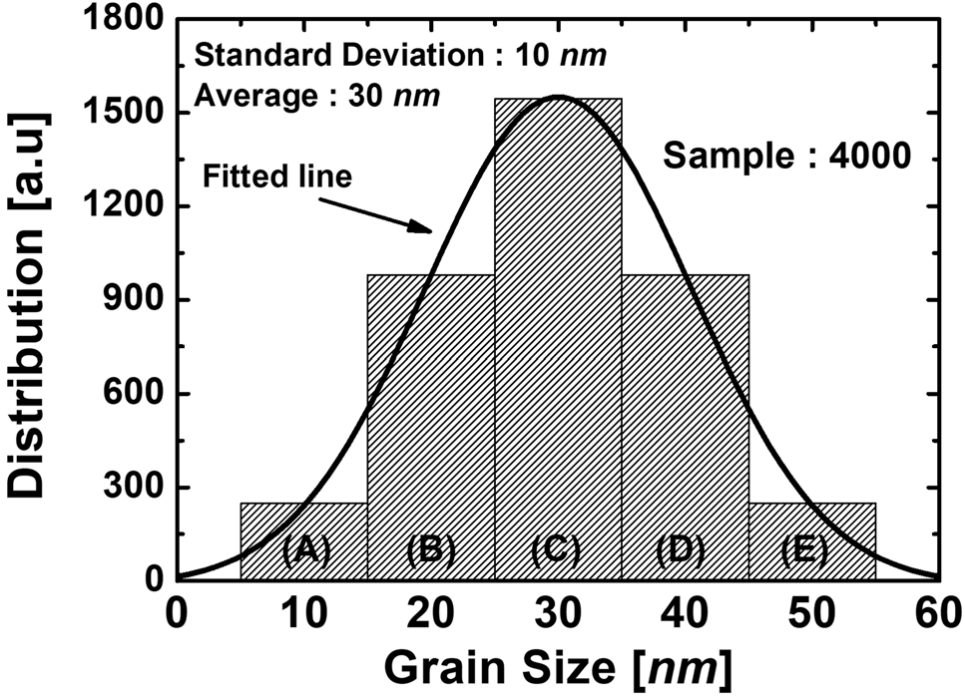
Histograms of the size of grains employed in the simulation work. The variations of the size of grains are generated from the Gaussian distribution. The average and SD for the Gaussian distribution are 30 nm and 10 nm, respectively.

The GB model, where a single GB exists in the channel region, is widely used. Also, most previous studies assumed that the *G*_size_ in the channel of poly-Si MOSFETs is the same when there are two or more than two GB in the channel region^5,12–14^. In this paper, we supposed *G*_size_ and its standard deviation (SD) are assumed to be 30 *nm* and 10 *nm*, respectively, to reflect realistic experimental results of *G*_size_ having a Gaussian distribution as shown in reference 13. Figure 2 shows the histogram of the *G*_size_ generated as assumed. As a result, TCAD simulations realistically represent variations in the size and the location of the GB in the transistor. The Box-Muller method, which generates a Gaussian distribution from uniformly distributed numbers, is the method for generating random GBs in the channel region. Furthermore, the trap distribution in the GBs of the poly-Si was calibrated using the experimental data in references 4 and 5 and assuming that all GBs contain the same amount of traps. The calibration results, including the GB trap distribution, are summarized in more detail in the Supplementary Information.

As shown in Fig. 2, the *G*_size_ distribution consists of groups (A), (B), (C), (D), and (E) depending on its size. As shown in Table 2, the *G*_*size*_s are set up as 5 nm to 15 nm, 15 nm to 25 nm, 25 nm to 35 nm, 35 nm to 45 nm and 45 nm to 55 nm, and the portions of each of the sizes are 6.2%, 24.5%, 38.6%, 24.5%, and 6.2%, respectively. If the Gaussian distributions for the *G*_*size*_s are applied to each channel of the proposed 3-D stacked ADG 1T-DRAMs, the number of samples becomes too large. Therefore, in this paper, the three channels of the proposed 3-D stacked 1T-DRAMs independently have one *G*_*size*_ among groups (A), (B), (C), (D), and (E) as shown in Table 3. For example, the group (ABC) in Table 3 means that the *G*_*size*_ of the channel in the bottom layer is 10 *nm*, the middle layer has a *G*_*size*_ of 20 *nm* and the top layer has a *G*_*size*_ of 30 nm. The probability multiplied by the 4000 samples and rounded-off is the value of the number of samples. To examine the electrical characteristics and the memory performances, the Sentaurus TCAD simulation tool is used^11^. To maximize the accuracy of the simulation works, various physical models are considered, including the Fermi–Dirac statistical model, the Shockley-Read-Hall (SRH) recombination model, the nonlocal band-to-band tunneling model, the trap-assisted-tunneling model, the Auger recombination model, the doping-dependent and field-dependent mobility models, the bandgap narrowing model, and the quantum confinement model^11^. The mobility models which are used for simulation are summarized in more detail in the Supplementary Information.

**Table 2:** The *G*_*size*_ of several cases in the single-layer ADG 1 T-DRAM of the proposed grain model: Group (A), (B), (C), (D), and (E).

Groups	*G*_size_	Probability (%)	Number of samples (4000 samples)
(A)	10 nm	6.2	248
(B)	20 nm	24.5	980
(C)	30 nm	38.6	1544
(D)	40 nm	24.5	980
(E)	50 nm	6.2	248

**Table 3:** The *G*_*size*_ of several cases in the 3-D stacked ADG 1 T-DRAM of the proposed GB model: from the group (AAA) to (EEE). A, B, C, D, and E means *G*_*size*_ is 10 nm, 20 nm, 30 nm, 40 nm, and 50 nm, respectively. The number of sample values is the probability multiplied by the 4000 and rounded up.

Groups	*Gsize* (Lower, middle, upper)	Probability (%)	Number of samples (4000 samples)
(AAA)	(10 nm, 10 nm, 10 nm)	0.024	1
(AAB)	(10 nm, 10 nm, 20 nm)	0.094	4
(AAC)	(10 nm, 10 nm, 30 nm)	0.148	6
(AAD)	(10 nm, 10 nm, 40 nm)	0.094	4
(AAE)	(10 nm, 10 nm, 50 nm)	0.024	1
(ABA)	(10 nm, 20 nm, 10 nm)	0.094	4
(ABB)	(10 nm, 20 nm, 20 nm)	0.372	15
⋮	⋮	⋮	⋮
(EEE)	(50 nm, 50 nm, 50 nm)	0.024	1

## Results and discussion

**Figure 3:**
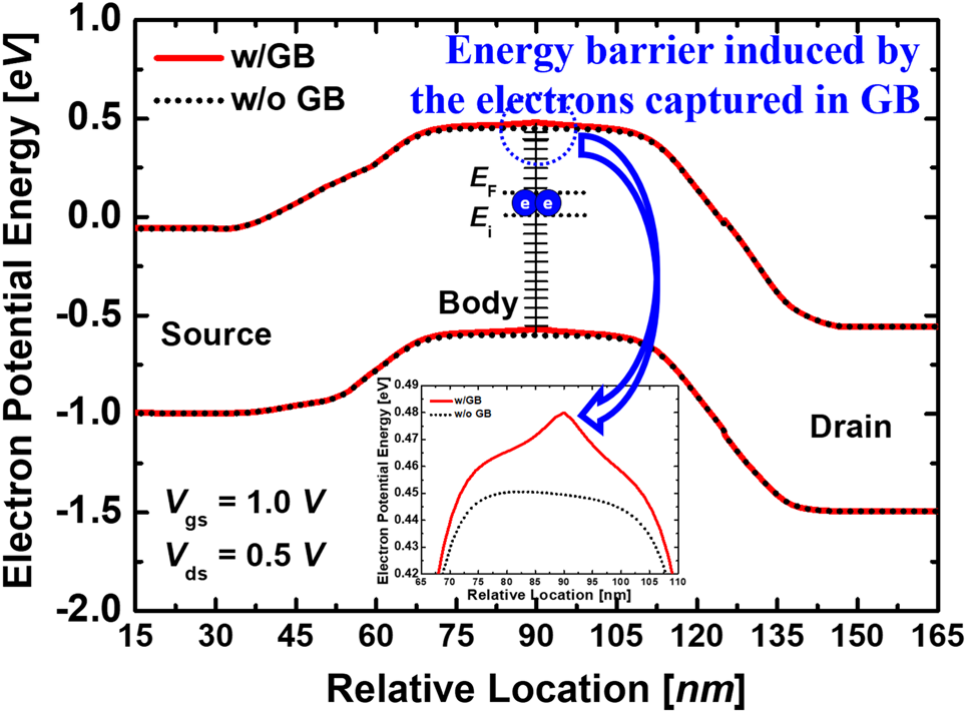
Electron potential energy of the proposed devices with and without GB at a read operation.

When the GB is located in the channel of the n-type MOSFET, the acceptor-like trap forms an energy barrier as electrons are trapped as shown in Fig. 3. The GB-induced potential barrier directly suppresses the current flow. As a result, it causes the *V*_th_ to rises^5^. Thus, it is shown that the variations in conduction characteristics are caused mainly by the number of GB located in the channel^3^. The *V*_*th*_ is defined by the constant-current method, which determines the *V*_th_ as the *V*_*g*_ value for *I*_*d*_ = 10^*−*7^ *A/µm*^15^.

### Statistical analysis of transfer characteristics

**Figure 4:**
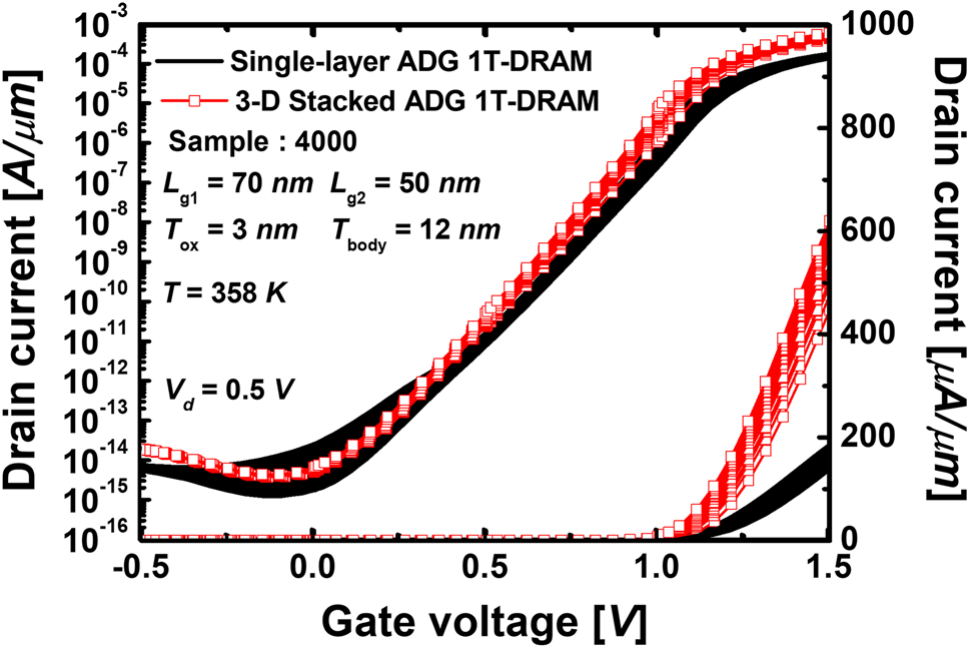
The *I*_*d*_–*V*_*g*_ curves of 4000 samples of the single-layer ADG 1 T-DRAMs and the 3-D stacked ADG 1 T-DRAMs. The parameters adopted are *L*_*mg*_ = 70 nm, *L*_*cg*_ = 50 nm, *T*_*ox*_ = 3 nm, *T*_*body*_ = 12 nm, and *T*_*spacer*_ = 30 nm.

Figure 4 shows the drain current (*I*_d_) versus the gate voltage (*V*_g_) curves of 4000 samples each single-layer ADG 1T-DRAMs and 3-D stacked ADG 1T-DRAMs with a *G*_size_ of 30 *nm* under drain voltage = 0.5 *V.* Histograms of (a) the *V*_*th*_s (b) the subthreshold swings (*SS*s) (c) the on-currents (*I*_*on*_s), and (d) the off-currents (*I*_off_s) obtained from simulations for 4000 samples with the proposed GB model, for proposed single-layer ADG 1T-DRAMs and 3-D stacked ADG 1T-DRAMs are extracted from Fig. 4.

**Figure 5:**
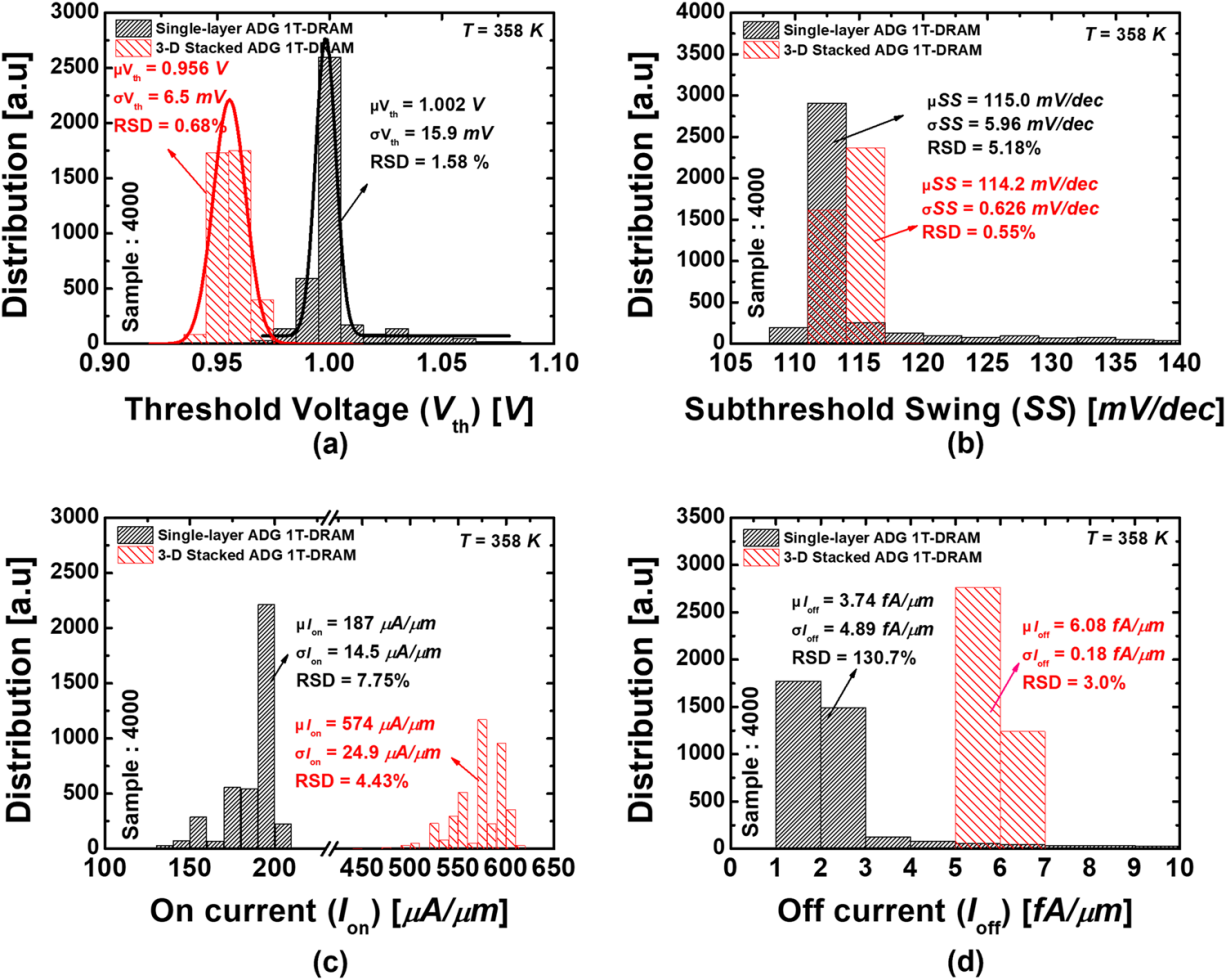
Histograms of (**a**) the *V*_*th*_s (b) the *SS*s (**c**) the *I*_*on*_s and (**d**) the *I*_*off*_ s obtained from the proposed single-layer 1 T-DRAMs’ and 3-D stacked 1 T-DRAMs’ 4000 samples with the proposed GB distribution.

In Fig. 5a, the averages of the extracted *V*_th_s of the single-layer ADG 1T-DRAMs and the 3-D stacked ADG 1T-DRAMs are 1.002 *V* and 0.956 *V*, respectively. The SDs for the *V*_th_s are 15.9 mV and 6.5 mV, respectively. The RSDs are 1.58% and 0.68%, respectively. The RSD is the SD divided by the mean and multiplied by 100. In other words, a large RSD indicates that the SD is large in comparison to the mean and it has a large dispersion. Since the RSD is expressed as a percentage, it is a coefficient of merit that can be used to compare the spread of data sets with different measurement units. The *I*_d_ are higher in the 3-D stacked ADG 1T-DRAMs due to the multi-layer structure, resulting in lower *V*_th_. The RSDs of the *V*_th_s are 1.58% for the single-layer ADG 1T-DRAMs and 0.68% for the 3-D stacked ADG 1T-DRAMs. Since the single-layer ADG 1T-DRAMs’ RSD is about two times than the 3-D stacked ADG 1T-DRAMs’ RSD. Therefore, the 3-D structure has better reliability for the *V*_th_ variations due to the GBs.

Figure 5b shows the distribution of *SS*s. The average of *SS*s of the single-layer ADG 1T-DRAMs and the 3-D stacked ADG 1T-DRAMs have similar values, 115.0 mv/dec and 114.2 mv/dec, respectively. The single-layer ADG 1T-DRAMs have the SSs ranging from 110 mv/dec to 140 mv/dec, as can be seen. However, in the case of the 3-D stacked ADG 1T-DRAMs, it can be seen that most of the *SS*s are distributed between 112 mv/dec and 117 mv/dec, respectively. This phenomenon occurs because they have 3-D stacked structures that complement the fluctuation in current characteristics caused by the GBs. This phenomenon is called the averaging effect. In the case of the single-layer ADG 1T-DRAM, it is significantly affected by the GBs of a channel region. However, even if one layer is significantly affected by the GBs, the performance variation in current and memory due to the GBs is small in the 3-D stacked ADG 1T-DRAM because the performance degradation is shared with the other two layers. This is a similar phenomenon that the decrease in work-function variation as the metal gate granularity decreases^16,17^. Consequently, the RSD of *SS*s of the 3-D stacked ADG 1T-DRAMs is more than 9 times smaller than the single-layer ADG 1T-DRAMs, which can be said to be excellent in the reliability of transfer characteristics.

Figure 5c shows the distribution of the *I*_*on*_s. The average *I*_*on*_s of the single-layer ADG 1T-DRAMs and the 3-D stacked ADG 1T-DRAMs are 1*.*87 × 10^*-*4^ A/µm and 5*.*74 × 10^*−*4^ A/µm, respectively. The average *I*_*on*_s of the 3-D stacked ADG 1T-DRAMs, which are multi-channel structures, is about three times larger than the single-layer ADG 1T-DRAMs’. A low *I*_*on*_ is a long-standing weakness of poly-Si transistors, but it can be solved using a 3-D stacked structure. In addition, the SDs of *I*_*on*_s are 1*.*45 × 10^*−*5^ A/µm and 2*.*49 × 10^*−*5^ A/µm, respectively. In respect to the SD, the dispersion of the 3-D stacked ADG 1T-DRAMs seems larger, but in the case of the RSD, which reflects the real dispersion, the single-layer ADG 1T-DRAMs’ RSD is 7.75% and the 3-D stacked ADG 1T-DRAMs’ RSD is 4.34%, respectively. In conclusion, the 3-D stacked 1T-DRAM has excellent transfer characteristics, such as a larger *I*_*on*_ and more minor variation.

Figure 5d shows the distribution of the *I*_*off*_ s. The average of *I*_*off*_ s of the 3-D stacked ADG 1T-DRAMs is 1.6 times larger than the single-layer ADG 1T-DRAMs’. The *I*_*off*_ s are the parameter with the most pronounced averaging effect. As aforementioned, thanks to the averaging effect, the RSD of the *I*_*off*_ of the 3-D stacked ADG 1T-DRAM is 3% owing to the 3-D structure. On the other hand, the transfer curves of the single-layer ADG 1T-DRAMs show large variances, which results in the RSD of 130.7% as shown in Fig. 4. The figure of merits (FOMs) of the transfer characteristics and the memory performances are summarized in Table 4.

**Table 4:** Comparison of the mean, the SDs, and the RSDs of the transfer characteristics and the memory performances of the single-layer ADG 1 T-DRAMs and the 3-D stacked ADG 1 T-DRAMs.

			Single-layer ADG 1 T-DRAM	3-D stacked ADG 1 T-DRAM
Transfer Characteristics	Threshold voltage (V_th_)	Mean	1.002 V	0.956 V
		SD	15.9 mV	6.5 mV
		RSD	1.58%	0.68%
	Subthreshold swing (SS)	Mean	115.0 mv/dec	114.2 mv/dec
		SD	5.96 mv/dec	0.626 mv/dec
		RSD	5.18%	0.55%
	On-current (I_on_)	Mean	1.87 × 10^−4^ A/µm	5.74 × 10^−4^ A/µm
		SD	1.45 × 10^−5^ A/µm	2.49 × 10^−5^ A/µm
		RSD	7.75%	4.43%
	Off-current (I_off_)	Mean	3.74 × 10^−15^ A/µm	6.08 × 10^−15^ A/µm
		SD	4.89 × 10^−15^ A/µm	1.825 × 10^−16^ A/µm
		RSD	130.7%	3.0%
Memory performances	Sensing margin (SM)	Mean	5.72 µA/µm	17.4 µA/µm
		SD	1.44 µA/µm	2.54 µA/µm
		RSD	25.2%	14.6%
	Retention time (RT)	Mean	212 ms	200 ms
		SD	116 ms	82 ms
		RSD	54.7%	41%

### Statistical analysis of memory performances

**Figure 6:**
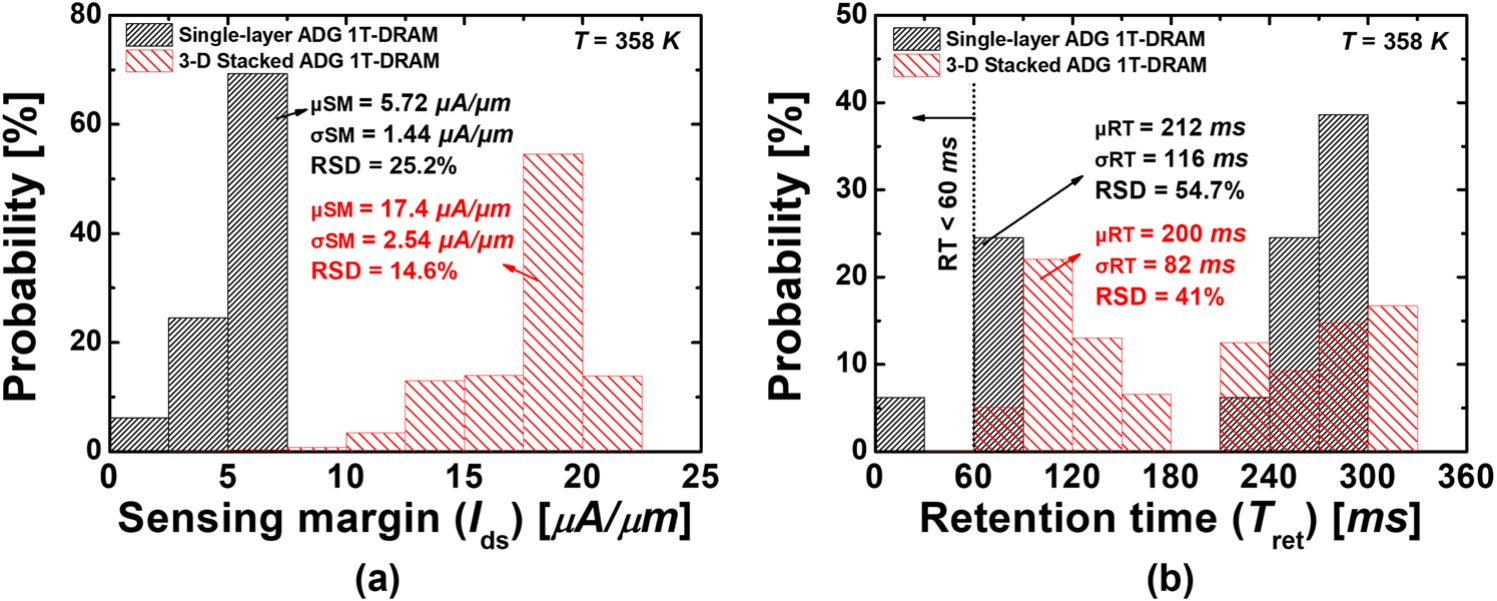
Histograms of (**a**) the SMs and (**b**) the RTs obtained from the proposed single-layer 1 T-DRAMs’ and 3-D stacked 1 T-DRAMs’ 4000 samples with the proposed GBs distribution.

Histograms of (a) the sensing margins (SMs) and (b) RTs from 4000 samples simulations with the proposed GB model, for the proposed single-layer ADG 1T-DRAMs and the 3-D stacked ADG 1T-DRAMs, are shown in Fig. 6a. In Fig. 6a, the averages of SMs of the single-layer ADG 1T-DRAM and the 3-D stacked ADG 1T-DRAM are 5.72 *µA/µm* and 17.4 µA/µm, the SDs are 1.44 µA/µm and 2.54 µA/µm and the RSDs are 25.2% and 14.6%, respectively. The SD of the SMs in the 3-D stacked ADG 1T-DRAMs is larger than the single-layers’, but the RSD is the opposite. Additionally, in the case of the single-layer ADG 1T-DRAMs, depending on the distribution of the GBs, some samples get poor SMs that are less than 3 µA/µm which is the reference value^18^. On the other hand, all samples of the 3-D stacked ADG 1T-DRAMs have superior to the SM of 3 *µA/µm*.

As shown in Fig. 6b, the average RTs of the single-layer ADG 1T-DRAMs and the 3-D stacked ADG 1T-DRAMs are 212 ms and 200 ms, respectively, showing similar values. However, as shown in Fig. 6b, the SDs of RTs are 116 ms and 82 ms and the RSDs of RTs are 54.7% and 41%. This indicates that the 3-D stacked ADG 1T-DRAMs have smaller RT deviations. In addition, in the single-layer ADG 1T-DRAMs, 6.2% of the samples are insufficient for RT to meet 64 ms which is the memory criteria of the international roadmap for devices and systems (IRDS) (>64 ms)^19^. However, in the case of the 3-D stacked ADG 1T-DRAMs, all the samples have longer RT than 60 ms as shown in Fig. 6b. In terms of the RT, the 3-D stacked ADG 1T-DRAMs are more reliable than the single-layer ADG 1T-DRAMs. The FOM of the memory performances is summarized in Table 4.

Consequently, when considering the SDs, and the RSDs of transfer characteristics and memory performances, the 3-D stacked ADG 1T-DRAMs showed excellent performance not only transfer characteristics but also memory performances. Moreover, they also show high immunity to the impact of GBs, especially on retention in the part of memory performances. Thus, our proposed 3-D stacked ADG 1T-DRAMs can be excellent devices in terms of reliability. Performance comparison of the conventional 1T-1C DRAMs, the capacitorless DRAMs, and the 3-D stacked asymmetric dual-gate 1T-DRAM are summarized in more detail in the Supplementary Information.

## Conclusion

In this work, a novel structure of 1 T-DRAM based on a poly-Si MOSFET transistor with the 3-D stacked ADG structure is designed and analyzed through a TCAD simulation. The optimized geometric parameters of the single-layer ADG 1 T-DRAM are used to perform the 3-D stacked ADG 1 T-DRAM^5^. The single-layer ADG 1 T-DRAM and the 3-D stacked ADG 1 T-DRAM are studied for their transfer characteristics and memory performances. Also, we statistically analyzed the reliability depending on the location and number of GBs. In the case of the single-layer ADG 1 T-DRAM, the RSDs of the *V*_*th*_, *SS, I*_on_, and *I*_off_ are 1.58%, 5.18%, 7.75%, and 130.7%, respectively. On the other hand, the RSDs of the *V*_*th*_, *I*_on_, *I*_off_, and *SS* of the 3-D stacked ADG 1 T-DRAMs are 0.68%, 0.55%, 4.43%, and 3.0%, respectively. The RSDs of the SM and RT of the single-layer ADG 1 T-DRAM are 25.2% and 54.7%, respectively. On the other hand, the RSDs of the SM and RT of the 3-D stacked ADG 1 T-DRAMs are 14.6% and 41%, respectively. The averaging effect occurs thanks to the 3-D stacked structure, which greatly helps to improve the reliability of the memory performances as well as the transfer characteristics of the 3-D stacked ADG 1 T-DRAM depending on the influence of GBs. As a result, when compared to the single-layer ADG 1 T-DRAM, the proposed novel 3-D stacked ADG 1 T-DRAM can implement a high reliable single memory cell.

## Supplementary Information

Supplementary Information 1.

Supplementary Information 2.

Supplementary Information 3.

Supplementary Information 4.

Supplementary Information 5.

Supplementary Information 6.

Supplementary Information 7.

## Data Availability

The datasets used and/or analysed during the current study available from the corresponding author on reasonable request.
