# Supplementary figures and images for: 3-D stacked polycrystalline-silicon-MOSFET-based capacitorless DRAM with superior immunity to grain-boundary’s influence
Source: Sci Rep. 2022 Aug 24;12:14455. doi: 10.1038/s41598-022-18682-y (PMC9402569; doi:10.1038/s41598-022-18682-y)

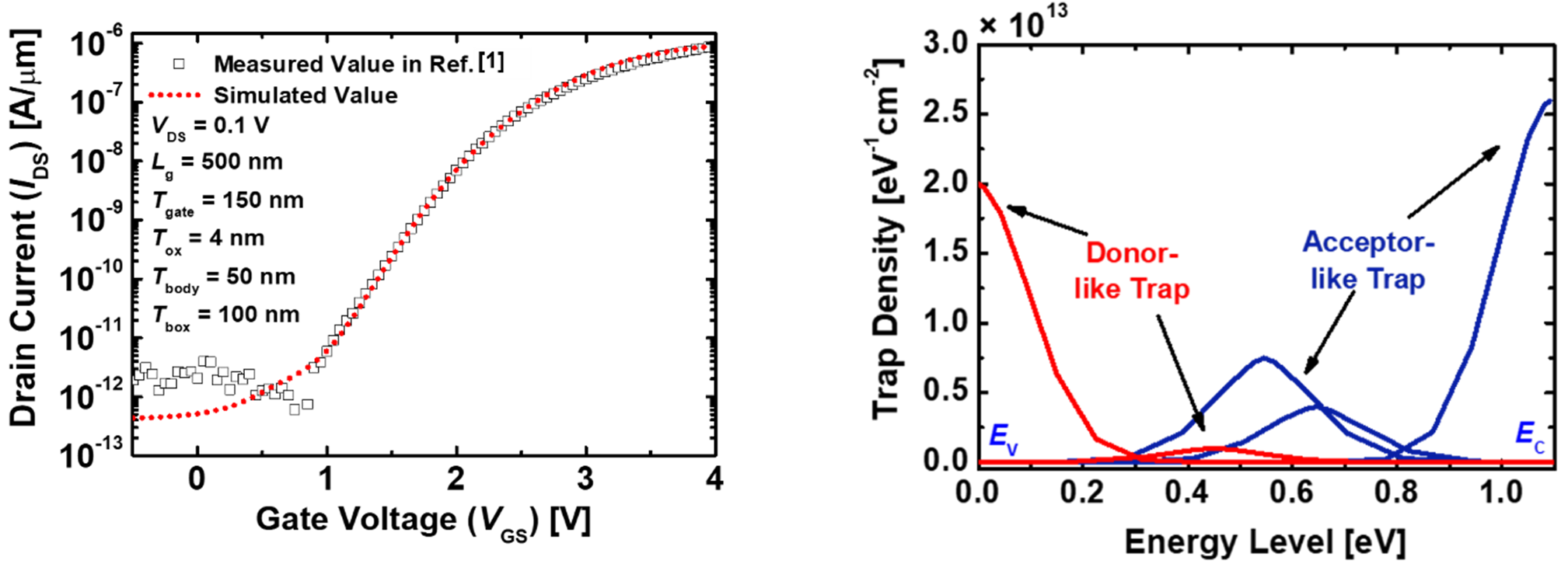

Supplement: Supplementary file 1 — Supplementary Information 1. [file 41598_2022_18682_MOESM1_ESM.png]

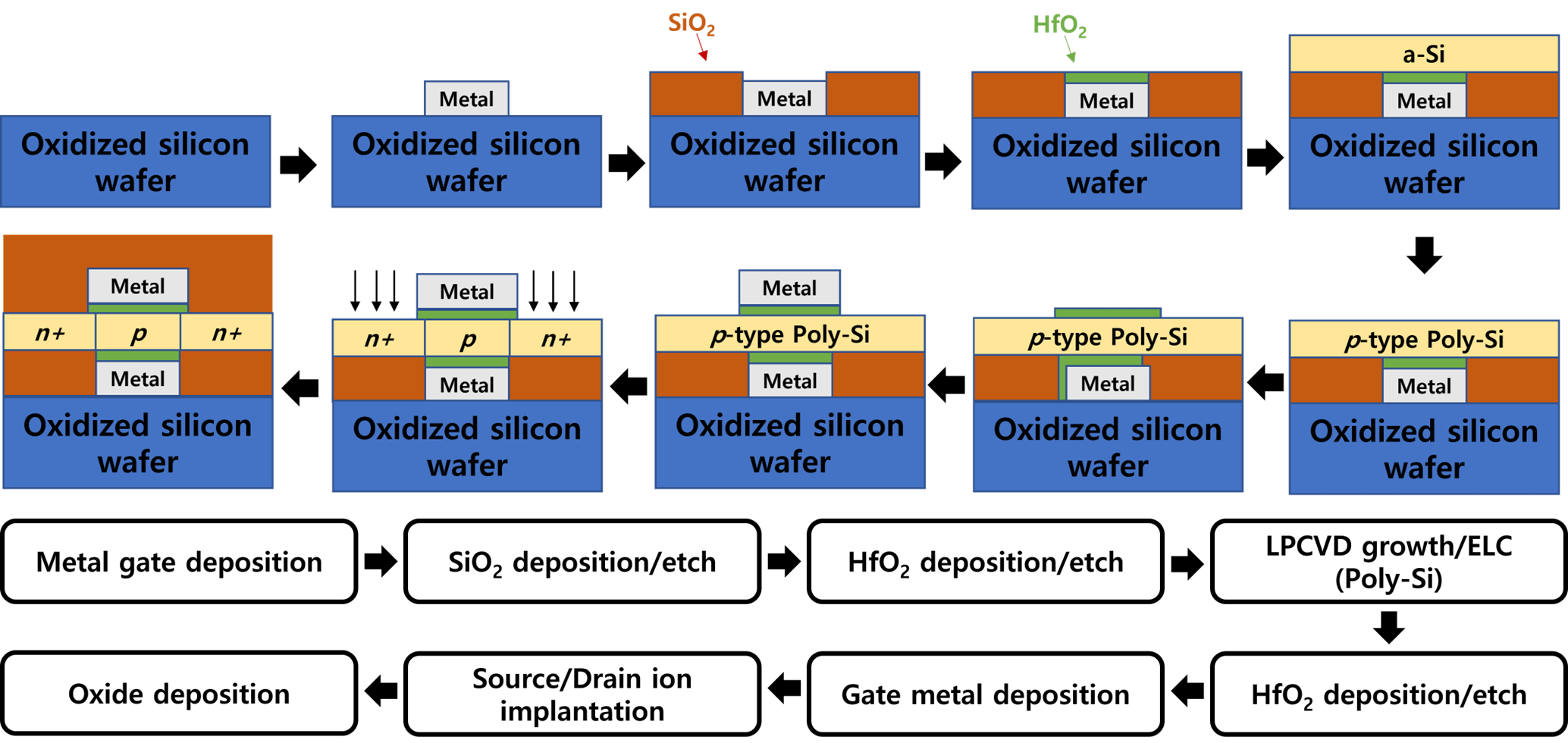

Supplement: Supplementary file 2 — Supplementary Information 2. [file 41598_2022_18682_MOESM2_ESM.png]

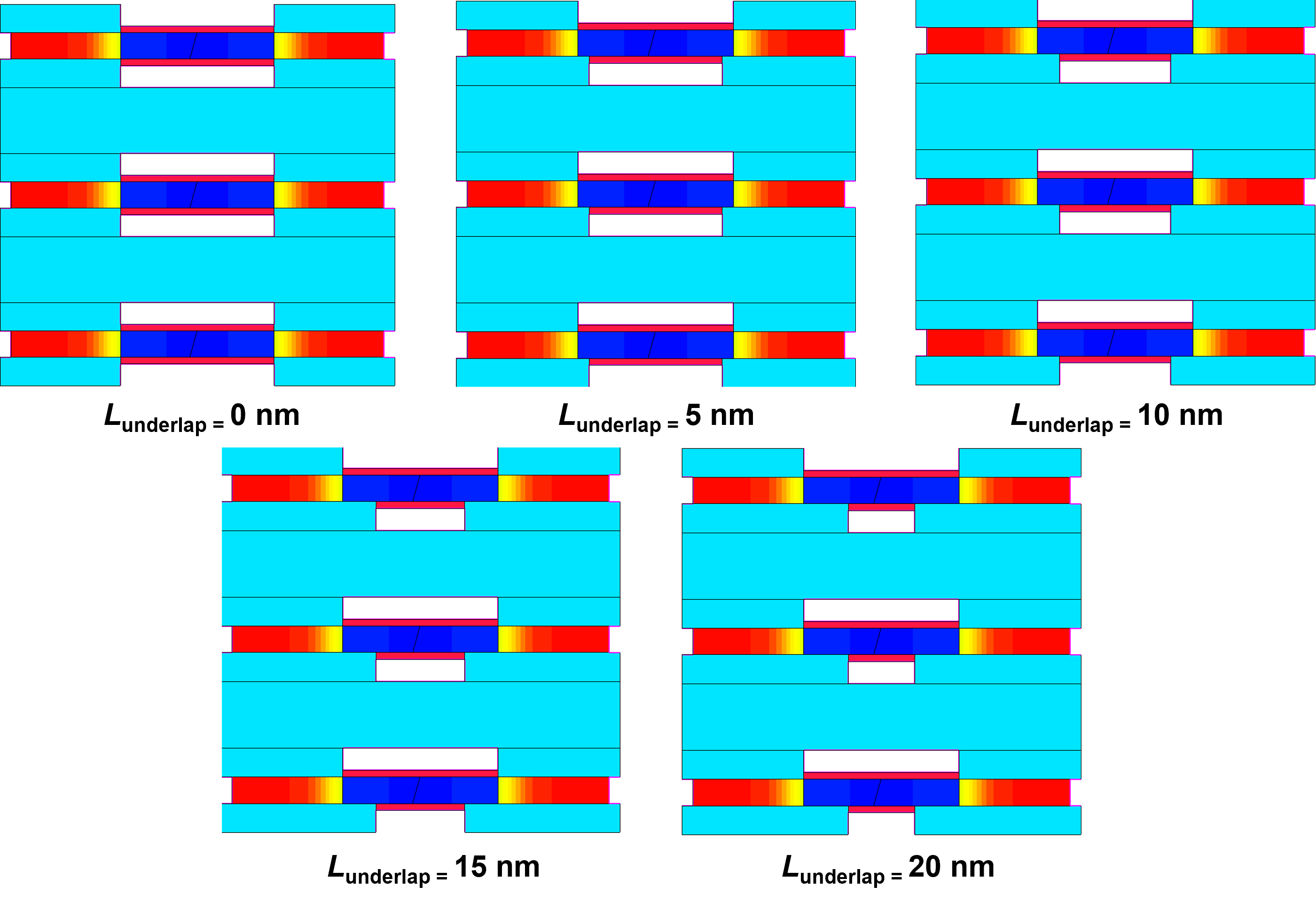

Supplement: Supplementary file 3 — Supplementary Information 3. [file 41598_2022_18682_MOESM3_ESM.png]

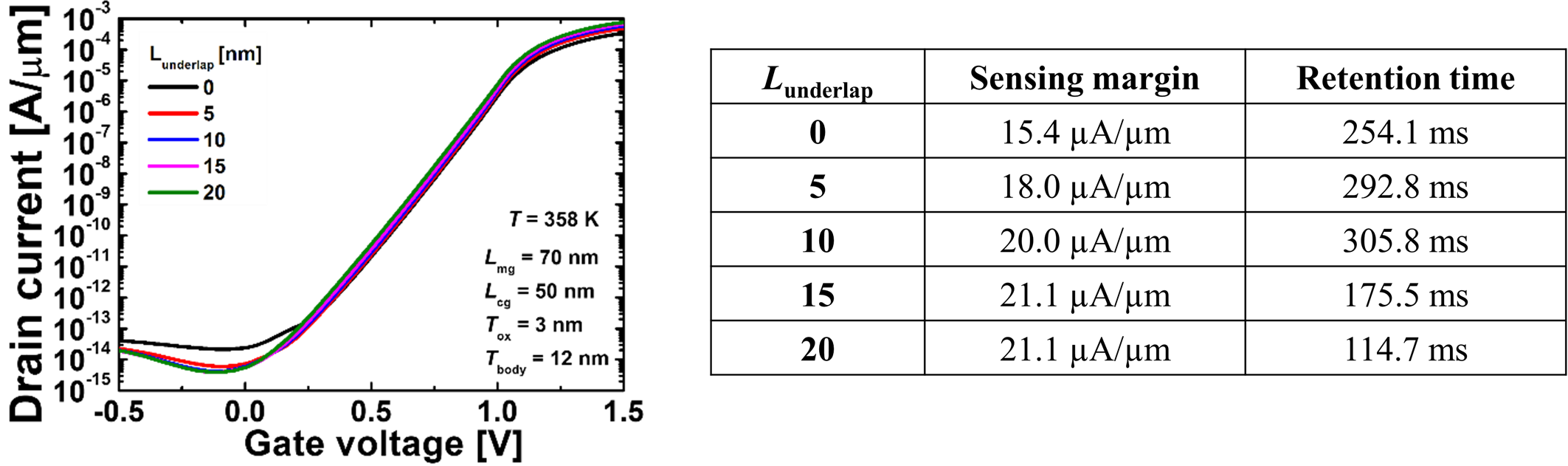

Supplement: Supplementary file 4 — Supplementary Information 4. [file 41598_2022_18682_MOESM4_ESM.png]

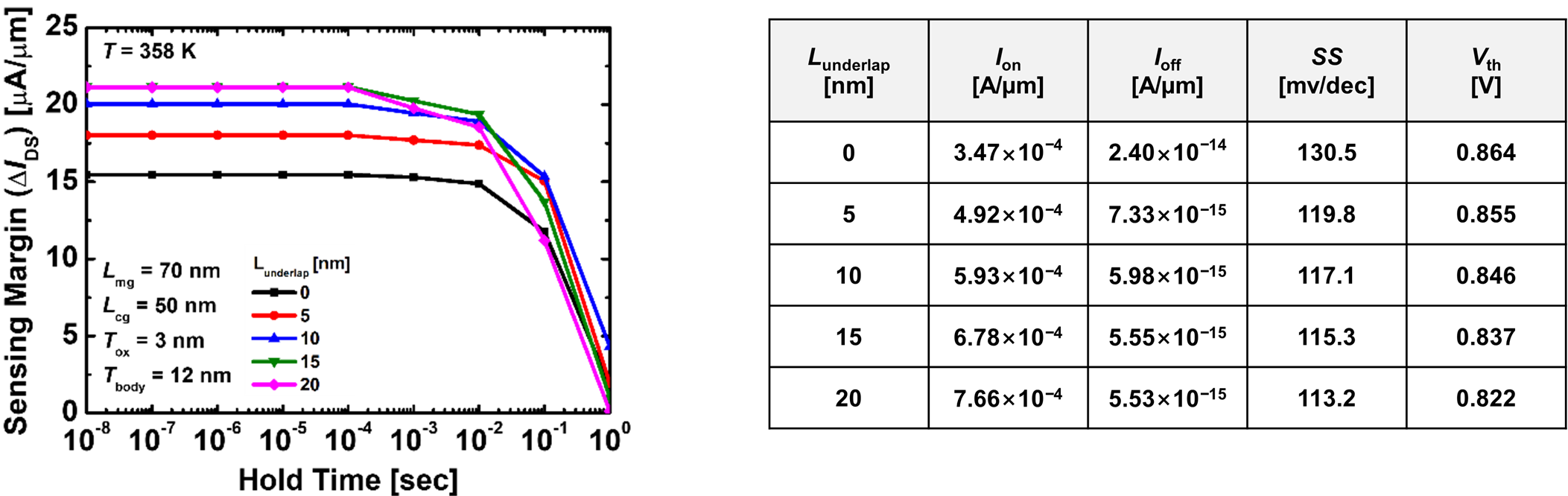

Supplement: Supplementary file 5 — Supplementary Information 5. [file 41598_2022_18682_MOESM5_ESM.png]
