# Supplementary material for: 3-D stacked polycrystalline-silicon-MOSFET-based capacitorless DRAM with superior immunity to grain-boundary’s influence
Source: Sci Rep. 2022 Aug 24;12:14455. doi: 10.1038/s41598-022-18682-y (PMC9402569; doi:10.1038/s41598-022-18682-y)
Supplement: Supplementary file 6 — Supplementary Information 6. [file 41598_2022_18682_MOESM6_ESM.docx]

Supplementary information for

**3-D stacked polycrystalline-silicon-MOSFET-based capacitorless DRAM with superior immunity to grain-boundary’s influence**

Sang Ho Lee, Jin Park, So Ra Min, Geon Uk Kim, Jaewon Jang, Jin-Hyuk Bae, Sin-Hyung Lee and In Man Kang*

Electronic and Electrical Engineering, Kyungpook National University, Daegu 702-201, South Korea

*imkang@ee.knu.ac.kr

# Calibration of the trap distribution in the GBs of the poly-Si

We calibrated the trap distribution with transfer characteristics from reference S1. For higher accuracy, a calibration that uses the experimental data in reference S1 was performed with various physical models, including trap density. As shown in Fig. S1 (a), the simulation results show a good agreement with the measured data using the calibrated parameters and the experimental data in reference S1. The trap distribution in the GB is illustrated in Fig. S1 (b).


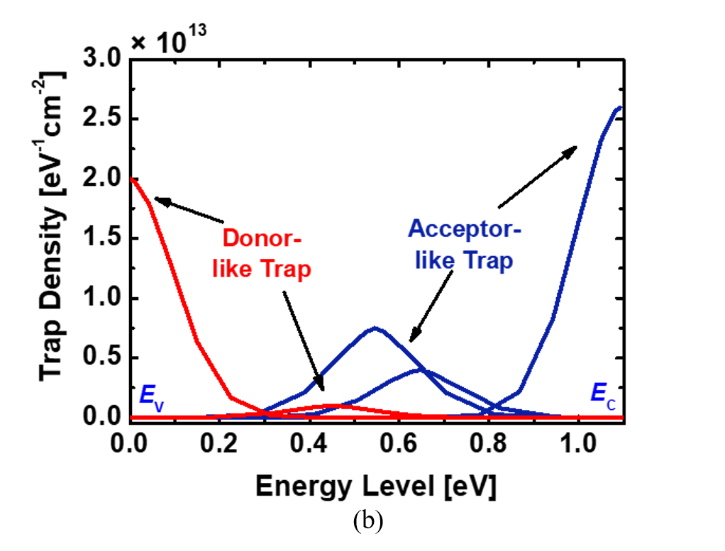

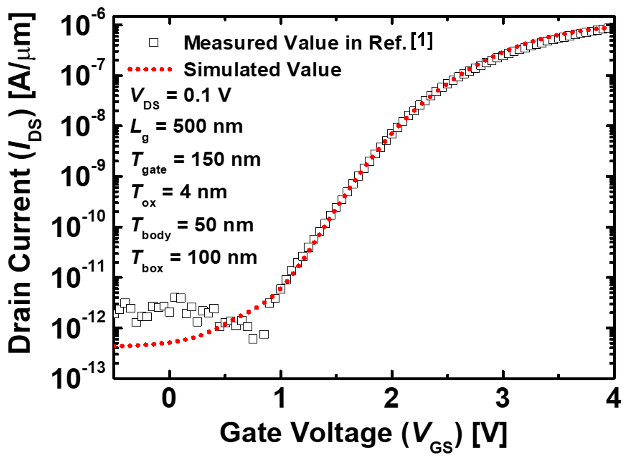
 (a) (b)

**Figure S1.** (a) Transfer characteristics of the simulation result with the calibrated parameters and the experimental data in reference S1. (b) GB trap distribution calibrated with the experimental data in reference S1.

In Fig. S1 (b), the GB had four trap states, depending on the energy level: acceptor-like shallow trap, acceptor-like deep trap, donor-like shallow trap, and donor-like deep trap. Therefore, two blue (acceptor-like trap) and red (donor-like trap) curves are in Fig. S1 (b).

# Mobility model used for simulating the capacitorless 1T-DRAM.

The Philips unified mobility model is applied in the simulation to calculate mobility precisely. This model unifies the descriptions of the bulk mobilities of the majority and minority carriers. In addition to the temperature-dependent factor, the model considers electron-hole scattering, screening of ionized impurities, and clustering of impurities. The Philips unified mobility model's phonon scattering and bulk scattering mechanisms contribute to carrier mobility. The combination of these factors contributes to the bulk mobility for each carrier according to Matthiessen’s rule:

$$\frac{1}{\mu_{i,b}}=\frac{1}{\mu_{i,L}}+\frac{1}{\mu_{i,DAeh}}$$

where *μ*_i,b_, *μ*_i,L_, and *μ*_i,DAeh_ are the bulk mobility, the mobility due to phonon scattering, and the mobility by all other bulk scatterings (scattering by the free carriers and ionized dopants), respectively.

#
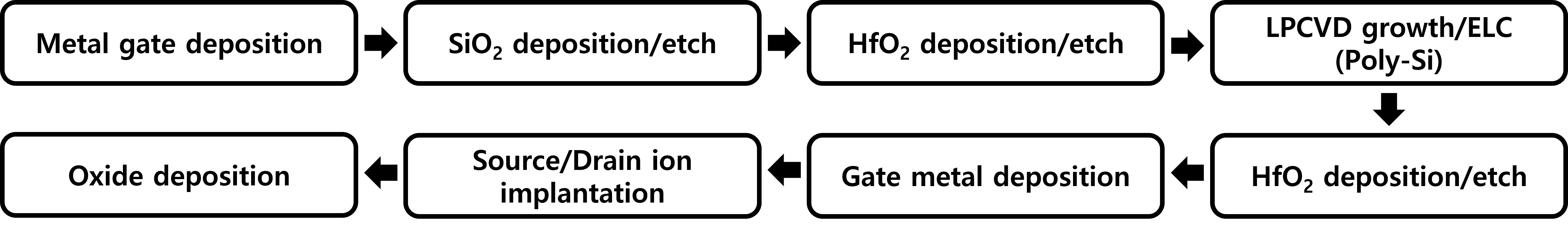

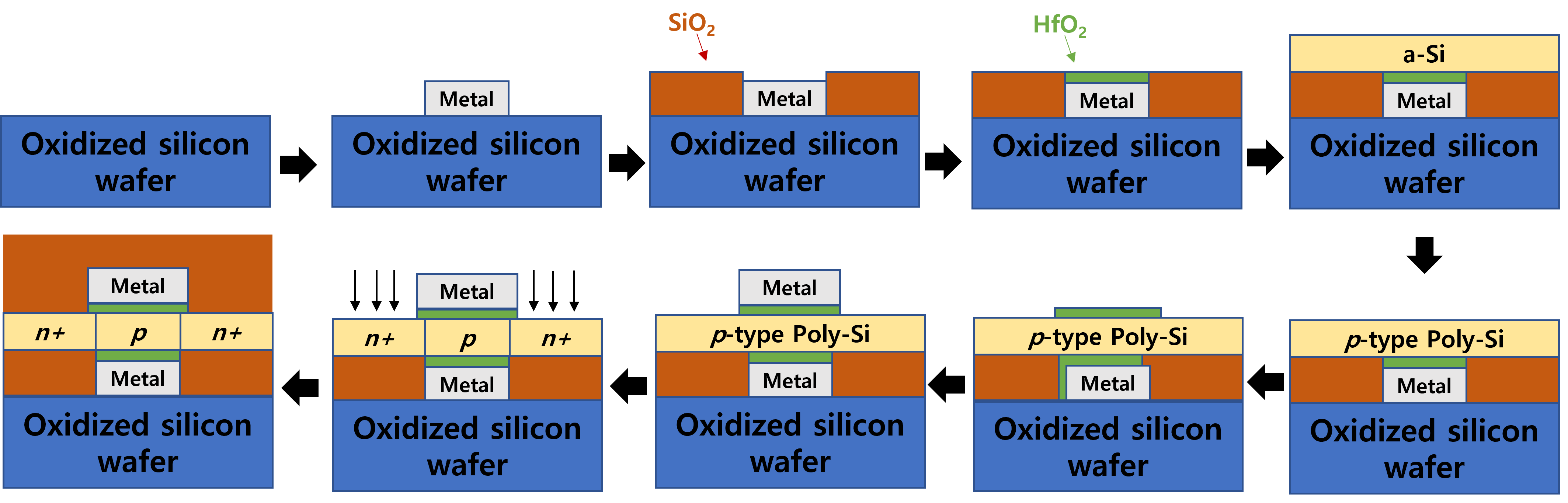
 Fabrication process flow for the 3-D stacked capacitorless DRAM.

**Figure S2.** Key fabrication steps for the proposed 3-D stacked 1T-DRAM

Figure S2 shows the key fabrication steps for the proposed 3-D stacked 1T-DRAM with asymmetric dual gate poly-Si MOSFET-based 1T-DRAM with crystallized via ELC. At first, the metal gate was deposited. After defining the control gate electrode, a SiO_2_ layer was deposited and etched. Then a HfO_2_ layer was deposited and etched. And an a-Si layer was deposited by LPCVD. Then, the samples were performed by excimer laser irradiation. After ELC, the HfO_2_ layer was deposited and etched. And then, the metal gate was deposited. Then, ion implantation was carried out to form the n-type source and drain. Afterward, an oxide layer was deposited. By repeating the above process, a stacked device can be implemented. Finally, the TSV process was carried out to connect between contacts of the 3-D stacked 1T-DRAM. The thermal budget was one of the significant challenges in implementing 3-D stacked transistors. The second layer and beyond of the device require high-temperature processing, which can threaten existing metallization materials or cause dopant diffusion in the lower layers of the device. These difficulties in the fabrication can be overcome by using excimer laser crystallization (ELC), and it can implement the 3-D stacked 1T-DRAM^S5^.

It is a little complex to implement 1T-DRAM for 3-D. However, poly-Si-based transistors have been employed in 3-D memory technology because of their significant advantages related to vertically stacking. Therefore, the proposed 1T-DRAM has the potential to implement high-density 3-D memory arrays in the future.

# Variation of underlap length in the proposed device


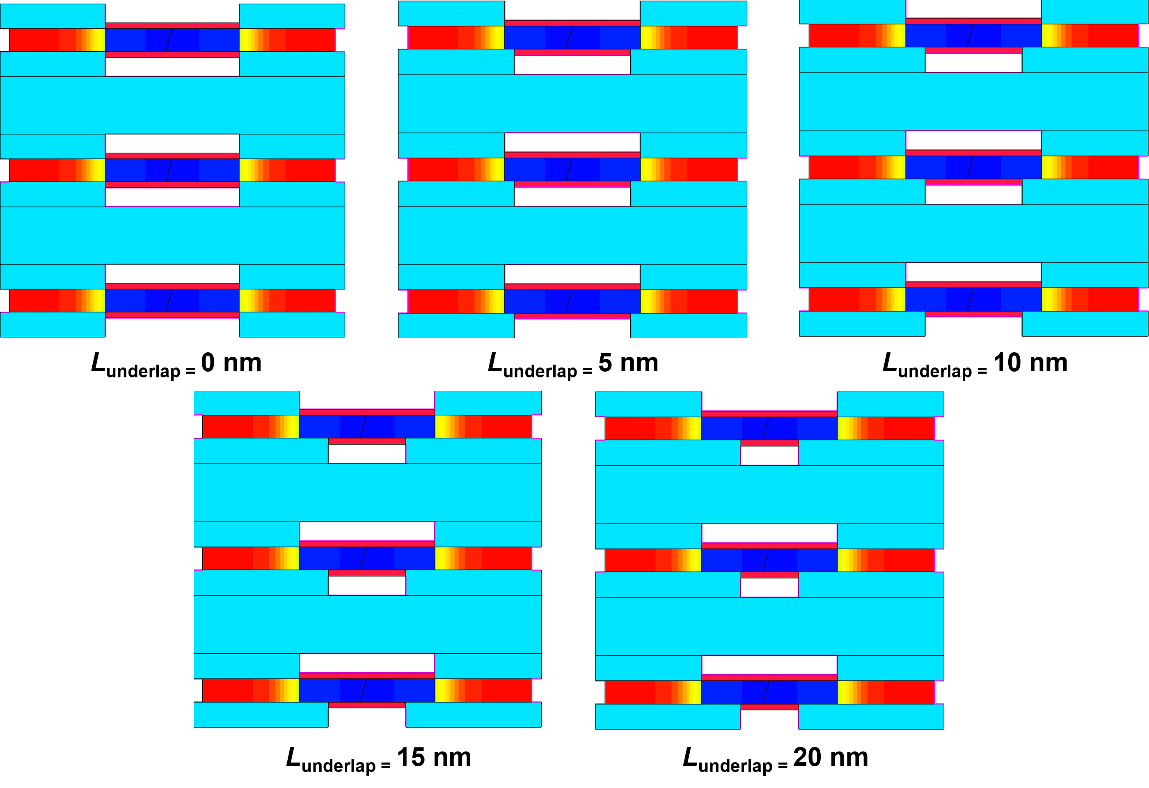


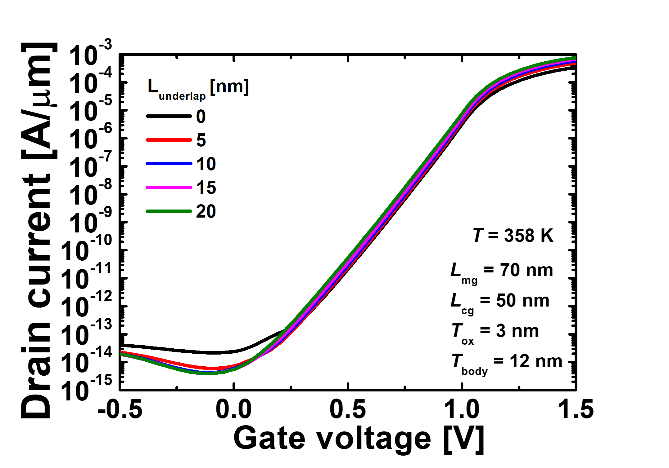
**Figure S3.** Contour map of the proposed capacitorless 1T-DRAM with different underlap length


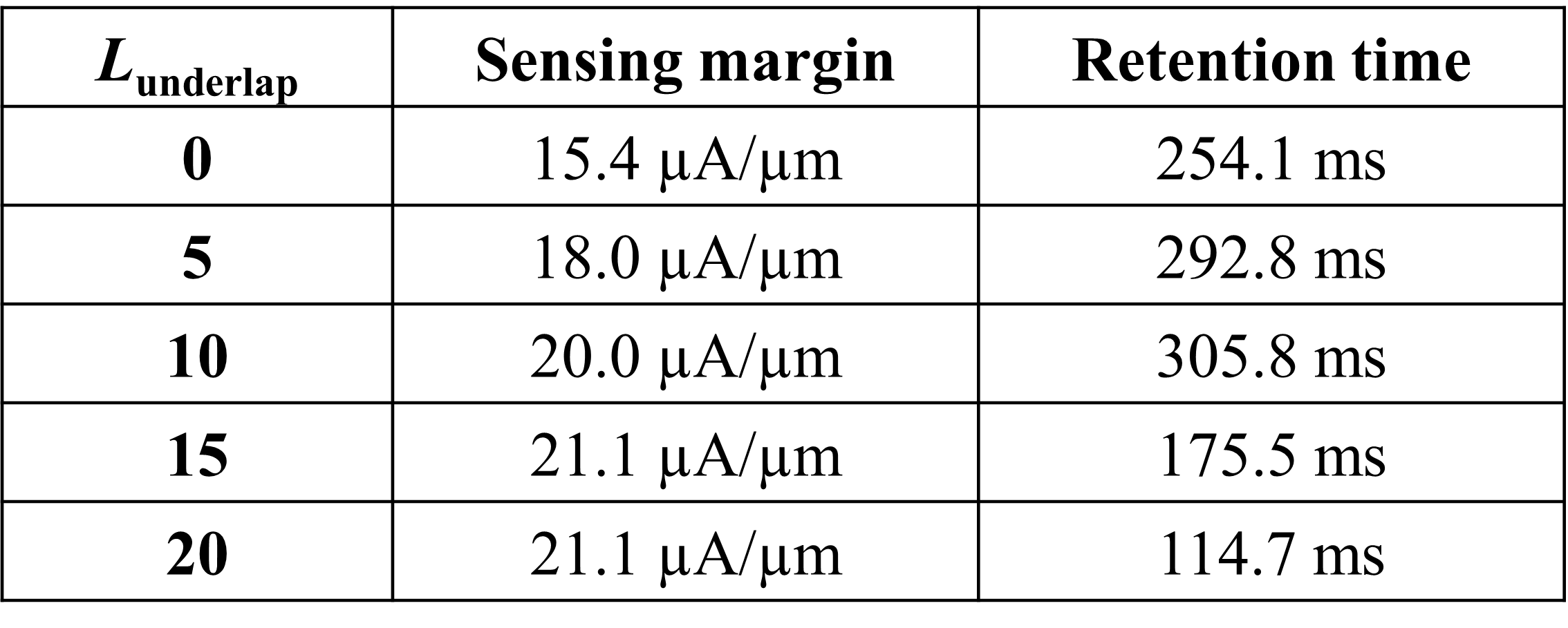


**Figure S4.** Transfer characteristics of the proposed capacitorless 1T-DRAM cell with different


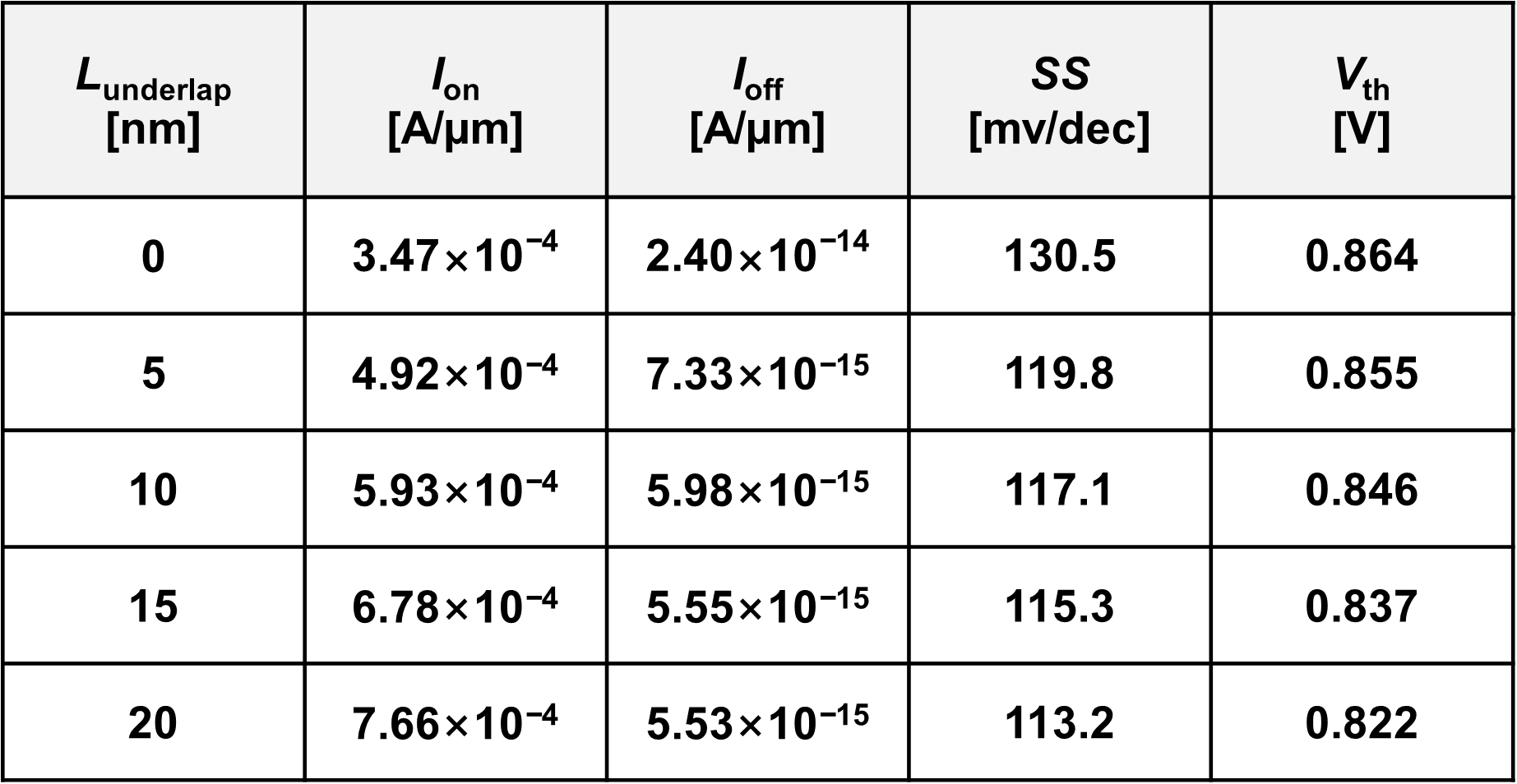

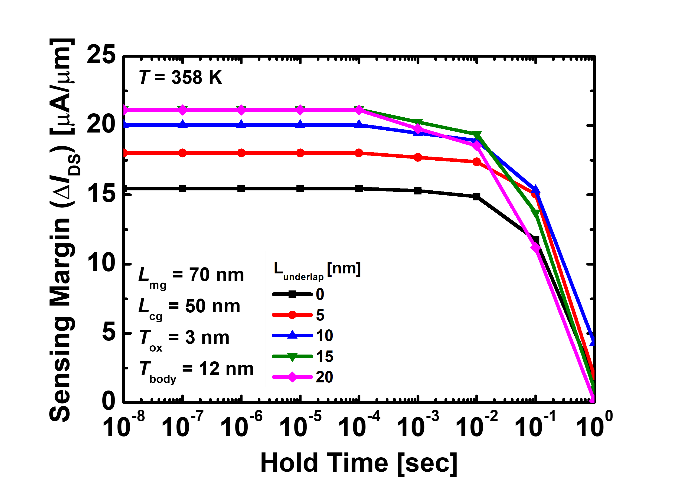
underlap length.

**Figure S5.** Memory performances of the proposed capacitorless 1T-DRAM cell with different underlap lengths

It is a little complex to implement 1T-DRAM for 3-D. However, poly-Si-based transistors have been employed in 3-D memory technology because of their significant advantages related to vertically stacking. Therefore, the proposed 1T-DRAM has the potential to implement high-density 3-D memory arrays in the future.

Thank you for your comment. We additionally simulated the proposed capacitorless 1T-DRAM with different underlap lengths (*L*_underlap_). As *L*_underlap_ increases, the influence of the control gate with high work-function decreases. This causes the depletion area of the channel to be reduced, and this results in an improvement in the transfer characteristics related to the sensing margin.

Consequently, the sensing margin is enhanced by increasing *L*_underlap_. However, the retention time increases up to *L*_underlap_ of 10 nm but then subsequently decreases. Retention time is affected not only by the generation/recombination rate but also by the number of holes. As *L*_underlap_ increases, the physical size of the quantum well that can store holes in the body region decreases. Additionally, the generation/recombination rate decreases as *L*_underlap_ increases. Below a *L*_underlap_ of 10 nm, the generation/recombination rate decreases and hole density decreases, but retention time increases because the number of holes is sufficient. When *L*_underlap_ is more than 10 nm, the generation/recombination rate also decreases; however, hole density in the body region is insufficient to cope with the decreasing number of holes by generation/recombination. As a result, when *L*_underlap_ is greater than 10 nm, the retention time decreases. It is described in more detail in reference S2.

# Performance comparison of conventional 1T-1C DRAM, capacitorless DRAM, and 3-D stacked asymmetric dual-gate 1T-DRAM

|  | Conventional 1T-1C DRAM | | | Capacitorless DRAM | | | This work |
| --- | --- | --- | --- | --- | --- | --- | --- |
|  | Ref.S6 | Ref.S7 | Ref.S8 | Ref.S9 | Ref.S10 | Ref.S11 |  |
| Sensing margin | *ΔV* =  100 mV | *ΔV =*  80 mV | *ΔV* =  100 mV | *ΔI* = 6.16 µA/µm | *ΔI* = 0.39 µA/µm | *ΔI* = 4.5 µA/µm | *ΔI* = 17.4 µA/µm |
| Retention time  @ *T* =358 K | 64 ms | 64 ms | 64 ms | 131ms | 10 ms | 11 ms | 200 ms |

**Table S1.** Performance comparison of conventional 1T-1C DRAM, capacitorless DRAM, and 3-D stacked asymmetric dual-gate 1T-DRAM

Table. S1 compares the performances of the conventional 1T-1C DRAMs, the capacitorless DRAMs, and 3-D stacked asymmetric dual-gate (ADG) 1T-DRAM. Conventional 1T-1C DRAM data is from references S6-S8 and capacitorless DRAM is from references S9-S11.

To operate the sense amplifier, conventional 1T-1C DRAM needs voltage differences in the 80~100 mV. For capacitorless DRAMs, 0.39 ~ 6.16 µA/µm is the reference value for sensing margin^S9-S11^. In this work, the mean value of SM of the 3-D stacked ADG 1T-DRAM is 17.4 μA/μm.

The conventional 1T-1C DRAM should meet the retention time of 64 ms, which is the memory criteria of the international roadmap for devices and systems (IRDS) (>64 ms)^S6-S8^. However, some capacitorless DRAMs have an RT value of less than 64 ms^S9-S10^. In this work, the mean value of RTs of the 3-D stacked ADG 1T-DRAMs is 200 ms. Additionally, all the samples have longer RT than 60 ms.

# References

S1. Seo, J. H. *et al.* Fabrication and characterization of a thin-body poly-si 1t dram with charge-trap effect. *IEEE Electron* *Device Lett.* 40, 566–569 (2019).

S2. Lee, S. H. *et al.* Polycrystalline-silicon-mosfet-based capacitorless dram with grain boundaries and its performances. *IEEE* *Access* 9, 50281–50290 (2021).

S3. Saadat, A., Van De Put, M. L., Edwards, H. & Vandenberghe, W. G. Channel length optimization for planar ldmos field-effect transistors for low-voltage power applications. *IEEE J. Electron Devices Soc.* 8, 711–715 (2020)

S4. Beohar, A, & Santosh K. V. Performance enhancement of asymmetrical underlap 3D‐cylindrical GAA‐TFET with low spacer width. *Micro & Nano Letters* 11, 443-445. (2016)

S5. Lee, I.-C. *et al*. High-performance vertically stacked bottom-gate and top-gate polycrystalline silicon thin-film transistors for three-dimensional integrated circuits. *Solid-state electronics* 77, 26–30 (2012)

S6. Sharroush, *et al*. Dynamic random-access memories without sense amplifiers. *e & i Elektrotechnik und Informationstechnik*, 129, 88-101. (2012).

S7. Birk, G. Method for multilevel DRAM sensing. *U.S. Patent*, 6 (2000)

S8. Okobiah, Oghenekarho, *et al.* Towards robust nano-CMOS sense amplifier design: a dual-threshold versus dual-oxide perspective. *Proceedings of the 21st edition of the great lakes symposium on Great lakes symposium on VLSI*. (2011).

S9. Yoon, Y. J. *et al.* Capacitor-less one-transistor dynamic random-access memory based on double-gate metal-oxide-semiconductor field-effect transistor with Si/SiGe heterojunction and underlap structure for improvement of sensing margin and retention time. *Journal of nanoscience and nanotechnology*, 19, 6023-6030 (2019)

S10. Yoon, Y. J. *et al*. One-transistor dynamic random-access memory based on gate-all-around junction-less field-effect transistor with a Si/SiGe heterostructure. *Electronics*, 9 2134, (2020)

S11. Ansari, Md Hasan Raza, et al. 1T-DRAM with shell-doped architecture*. IEEE Transactions on Electron Devices,* 66, 428-435. (2018).
