# Supplementary material for: 3-D stacked polycrystalline-silicon-MOSFET-based capacitorless DRAM with superior immunity to grain-boundary’s influence
Source: Sci Rep. 2022 Aug 24;12:14455. doi: 10.1038/s41598-022-18682-y (PMC9402569; doi:10.1038/s41598-022-18682-y)
Supplement: Supplementary file 7 — Supplementary Information 7. [file 41598_2022_18682_MOESM7_ESM.docx]

|  | Conventional 1T-1C DRAM | | | Capacitorless DRAM | | | This work |
| --- | --- | --- | --- | --- | --- | --- | --- |
|  | Ref.S6 | Ref.S7 | Ref.S8 | Ref.S9 | Ref.S10 | Ref.S11 |  |
| Sensing margin | *ΔV* =  100 mV | *ΔV =*  80 mV | *ΔV* =  100 mV | *ΔI* = 6.16 µA/µm | *ΔI* = 0.39 µA/µm | *ΔI* = 4.5 µA/µm | *ΔI* = 17.4 µA/µm |
| Retention time  @ *T* =358 K | 64 ms | 64 ms | 64 ms | 131ms | 10 ms | 11 ms | 200 ms |

**Table S1.** Performance comparison of conventional 1T-1C DRAM, capacitorless DRAM, and 3-D stacked asymmetric dual-gate 1T-DRAM
